# Supplementary material for: Highly Efficient A-to-G Editing in PFFs via Multiple ABEs
Source: Genes (Basel). 2023 Apr 13;14(4):908. doi: 10.3390/genes14040908 (PMC10137487; doi:10.3390/genes14040908)
Supplement: Supplementary file 1 [file genes-14-00908-s001.zip › genes-2248170-supplementary.pdf]

Supplementary information

Highly efficient A-to-G editing in PFFs via multiple ABE

Qiqi Jing[1], Haoyun Jiang[1], Yaya Liao[1], Qiang Yang[1], Weiwei Liu [1*], Yuyun Xing [1*]

[1]State Key Laboratory of Pig Genetic Improvement and Production Technology, Jiangxi Agricultural University, Nanchang 330045, China

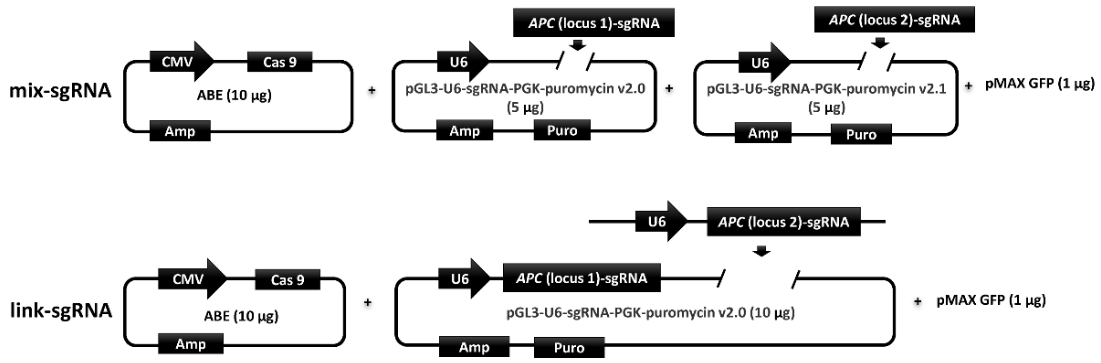

Figure S1: The schematic diagrams of mix-sgRNA and link-sgRNA strategies.

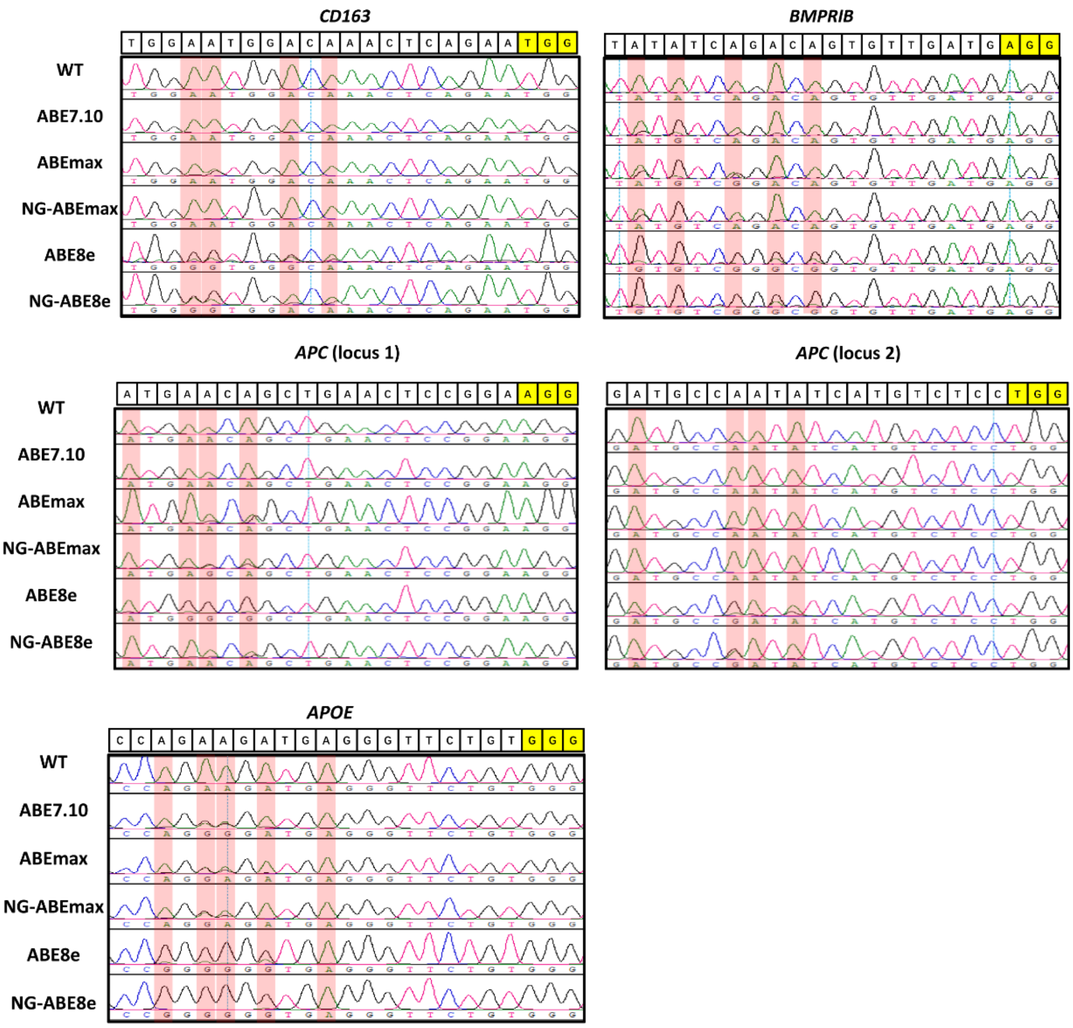

Figure S2: Representative sanger sequencing chromatograms of PFFs transfected with ABEs targeting *CD163*, *BMPRIB*, *APC* and *APOE* loci.

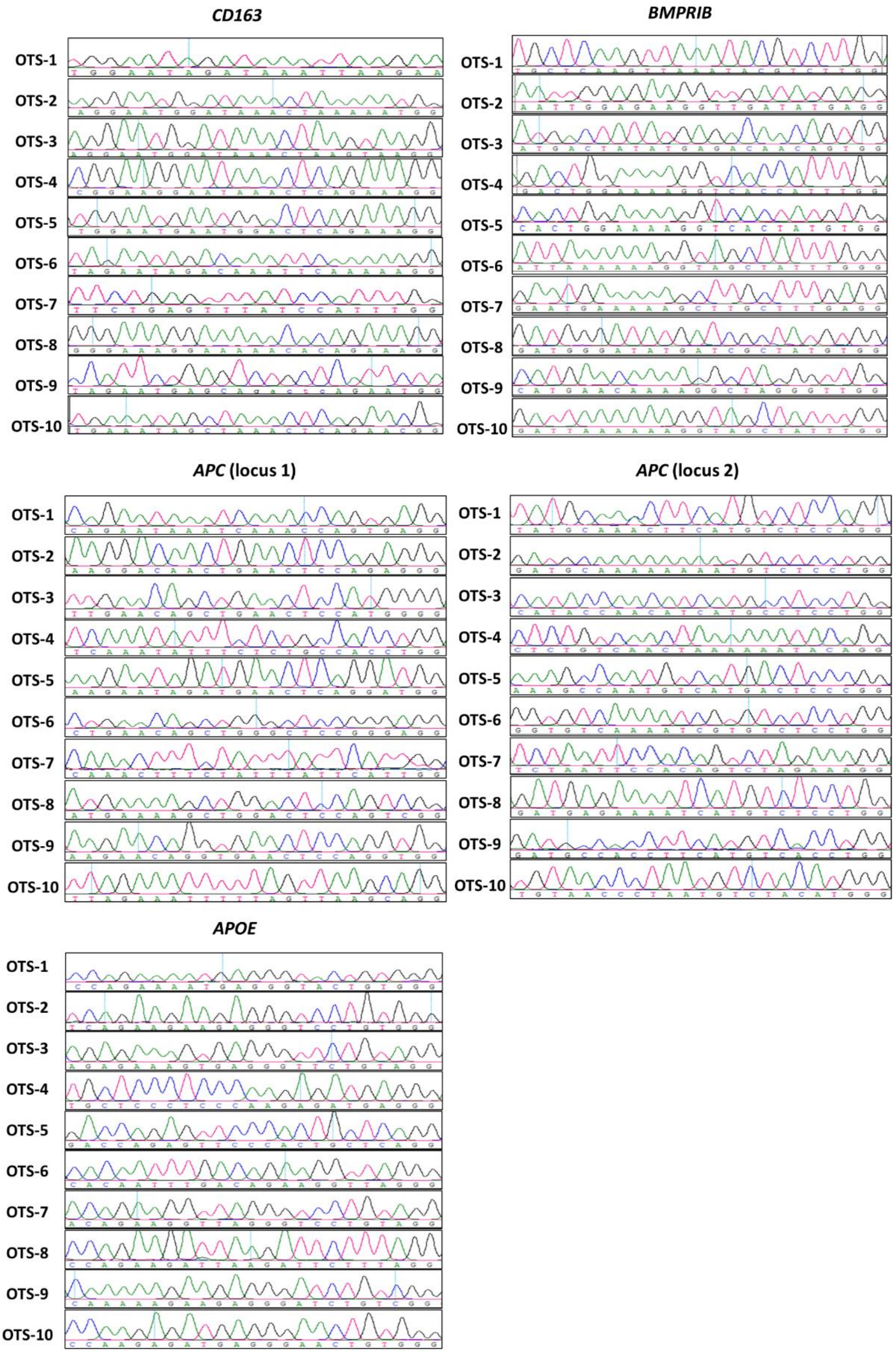

Figure S3: Representative sequencing chromatograms of top 10 predicted OTS for each sgRNA.

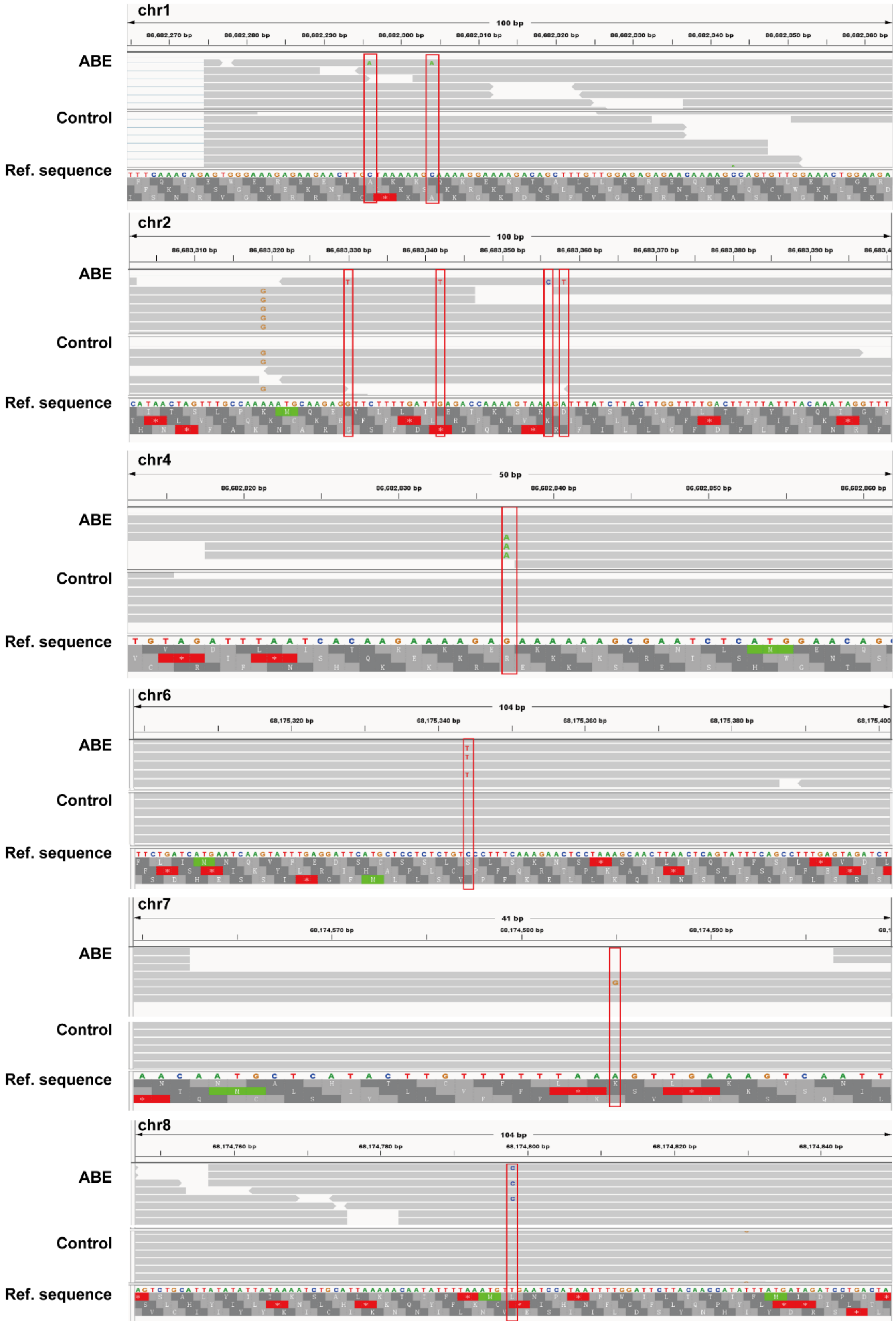

Figure S4: Comparison situation of RNA editing sites between RNA-seq reads and reference genome sequence. The red box represents the RNA editing sites identified.

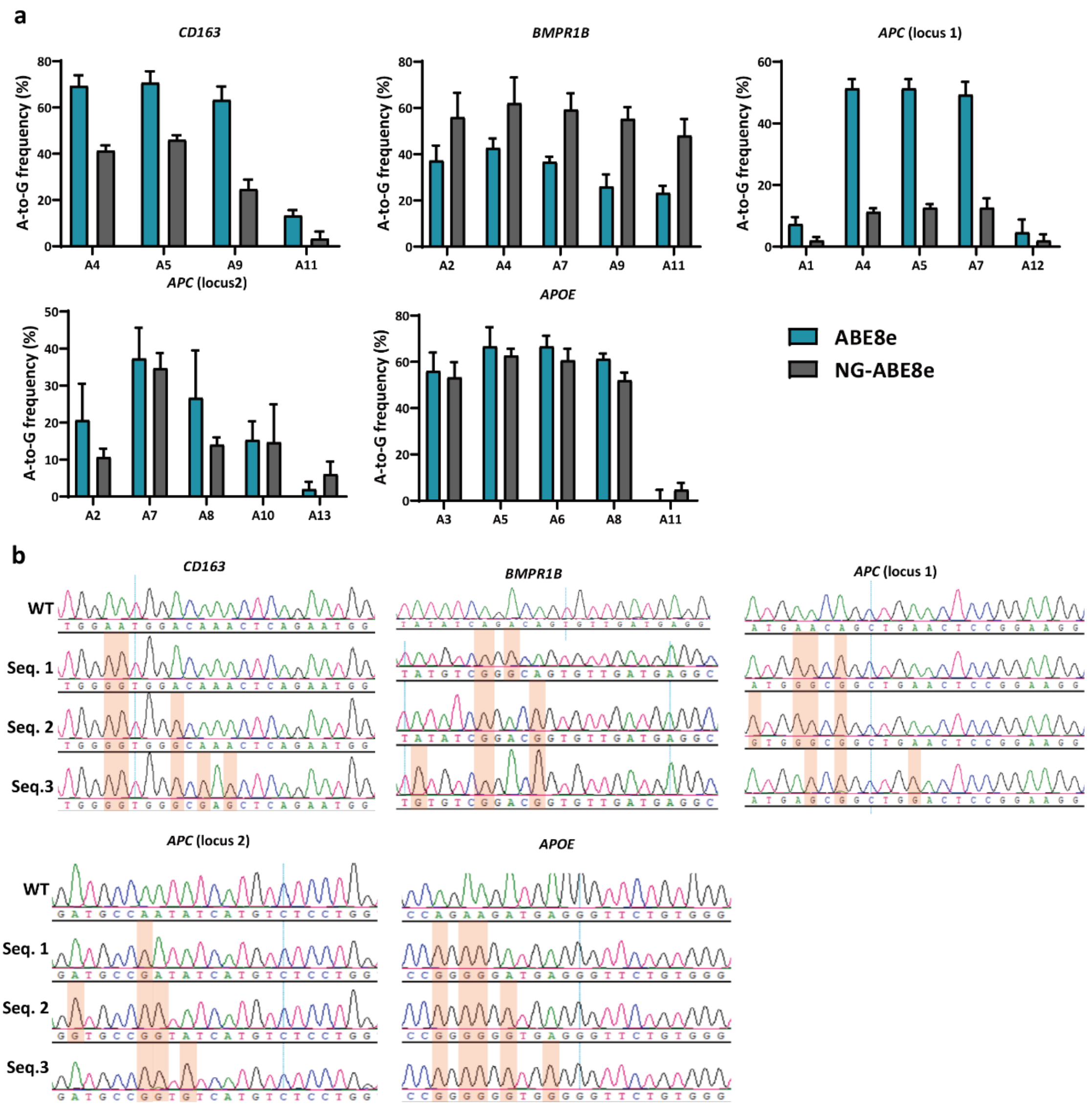

Figure S5: ABE-mediated A-to-G conversion using ABE8e and NG-ABE8e editors without puromycin selection in PFFs. (a) A-to-G conversion efficiencies at 5 targeting loci. n=3, independent replicates. (b) Representative TA cloning-based sequencing chromatograms of PFFs transfected with ABE8e editor targeting 5 loci.

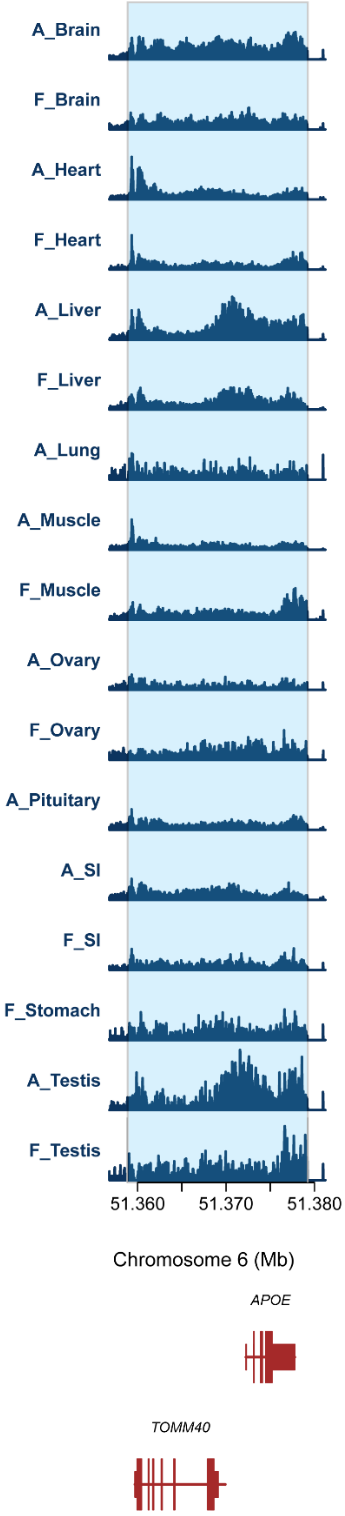

Figure S6: Distribution of H3K27ac modification in chromosome 6. Shaded areas indicate the H3K27ac modification. A: Adult; F: Fetal.
